# Classification of Nonallergic Rhinitis Syndromes With a Focus on Vasomotor Rhinitis, Proposed to be Known henceforth as Nonallergic Rhinopathy

**DOI:** 10.1097/WOX.0b013e3181a9d55b

**Published:** 2009-06-15

**Authors:** Michael A Kaliner

**Affiliations:** 1 From the Institute for Asthma and Allergy, Chevy Chase, Md, and George Washington University School of Medicine, Washington, DC

**Keywords:** rhinitis, vasomotor rhinitis, allergic rhinitis, nonallergic rhinitis, nonallergic rhinopathy

## Abstract

Many patients have nasal syndromes that are nonallergic and noninfectious and not caused by mechanical or anatomic abnormalities. There are at least 8 recognized nonallergic rhinitis syndromes: drug-induced rhinitis including rhinitis medicamentosa, gustatory rhinitis, hormonally induced rhinitis including the rhinitis of pregnancy, nonallergic rhinitis with eosinophilia syndrome, senile rhinitis, atrophic rhinitis, cerebral spinal fluid leak, and vasomotor rhinitis. Few studies have explored etiologic causes. These syndromes are distinguished by clinical characteristics, recognized triggering conditions, and concomitant observations such as nasal eosinophilia or cerebral spinal fluid leak. Until more specific subjective clinical characteristics and/or objective measures can better define and differentiate underlying causes for these disparate diseases, they will remain a murky group of overlapping syndromes.

## Introduction

The generally accepted universal clinical characteristics of the diseases classified within the category of chronic nonallergic rhinitis (NAR) syndromes include only the following: (1) nasal symptoms and (2) no evidence of concomitant allergic disease as determined by negative skin prick testing for relevant allergens and/or negative allergen-specific antibody tests. This classification automatically excludes infectious rhinitis, rhinosinusitis, and mechanical/anatomical abnormalities as causes of the chronic symptoms.

There are at least 8 separate clinical entities that can be classified among the disorders that make up the NAR syndromes (Table 1), with vasomotor rhinitis (VMR) being the clinically most common and important one[1,2]. By some estimates, allergic rhinitis (AR) affects up to 58 million Americans, whereas NAR affects more than 19 to 30 million patients in the United States[1,3,4]. In these estimates of prevalence, VMR is the most prevalent of the NAR syndromes. The cost of care for allergic rhinitis in the United States is estimated at more than $5 to 6 billion annually[5,6]. It is estimated that VMR accounts for $2 to 3 billion[1,2]. Hard data on the incidence and frequency of NAR subtypes are limited. However, it is recognized that VMR is the most prevalent type of NAR, affecting an estimated 37% to 61% of patients diagnosed with rhinitis[7]. In 1 survey of US medical practices, a prospective classification of 2500 patients with rhinitis was performed, and it was found that 43% of the patients had "pure" AR, 23% had pure VMR, and 34% had rhinitis with both AR and VMR features (sometimes known as mixed rhinitis)[7,8]. These data suggest that at least 57% of rhinitis patients have some contribution from NAR causing their rhinitis symptoms. Similar European studies have found that approximately 1 in 4 patients complaining of nasal symptoms has "pure" NAR[9]. Recent estimates suggest that 50 million Europeans have NAR, with a total prevalence of more than 200 million worldwide[10].

**Table 1:** Chronic Rhinitis Syndromes that are Nonallergic and Noninfectious and Not Due to Anatomical/Mechanical Causes*

• Drug-induced rhinitis, including rhinitis medicamentosa
• Gustatory rhinitis
• Hormonal-induced rhinitis, including the rhinitis of pregnancy
• Nonallergic rhinitis with eosinophilia
• Senile rhinitis
• Atrophic rhinitis
• Cerebral spinal fluid leak
• Nonallergic rhinopathy (NAR), previously known as vasomotor rhinitis (VMR), or idiopathic nonallergic rhinitis

*Both anatomic/mechanical abnormalities and chronic infectious rhinitis and rhinosinusitis are excluded.

Adapted from *J World Allergy Org*. 2009;2:20-25 and *J Allergy Clin Immunol*. 2008;122(2 suppl):S1-S84.

## Classification of NAR syndromes

There have been several recent attempts at classifying the chronic nasal syndromes not associated with allergic disease[2,11,12]. The reader is referred to these references for additional approaches to this problem and for a more complete bibliography[12]. The approach taken herein combines the overall recommendations from these 3 sources, combined with input from other experts at a consensus conference from which this series of papers is derived.

There are at least 8 subtypes that fill the criteria for nonallergic rhinitis (Table 1): drug-induced rhinitis, gustatory rhinitis (rhinorrhea associated with eating, especially hot and spicy foods), hormonally induced rhinitis, nonallergic rhinitis with eosinophilia syndrome (NARES), senile rhinitis, atrophic rhinitis, cerebral spinal fluid (CSF) leak, and vasomotor rhinitis (VMR)[2]. The 2 nonallergic processes, infectious rhinitis (including bacterial rhinitis and chronic rhinosinusitis) and mechanical/anatomical abnormalities, are excluded from this classification.

Hormonally induced rhinitis reflects responses to endogenous female hormones. The rhinitis of pregnancy is an extremely common condition, affecting up to 20% to 30% of pregnancies, and particularly notable during the last trimester[13]. It typically resolves spontaneously within 2 weeks of delivery. As 1 clue to specific causes, Ellegard showed that women with rhinitis of pregnancy had elevated serum placental growth hormone levels compared to pregnant women without rhinitis[14]. However, it is usually assumed that the rhinitis of pregnancy reflects the mucosal engorgement found in the last trimester as a consequence of progesterone stimulation. Thus, the nasal mucosa becomes engorged and congestion ensues, as all of the mucous membranes are affected by the hormonal changes of pregnancy[15]. Some patients develop similar symptoms premenstrually on a cyclical basis[15].

Drug-induced rhinitis includes rhinitis medicamentosa, which is the descriptive name for the nasal congestion and rebound rhinitis caused by repeated administration of topical nasal decongestants. The most common cause of rhinitis medicamentosa is overuse of topical nasal decongestants such as oxymetazoline or phenylephrine. When used briefly (less than 3-5 days consecutively), these medications provide significant relief of nasal congestion. However, with chronic use, rebound nasal congestion can occur and can be quite severe. The exact mechanism is poorly understood but theories suggest involvement of recurrent nasal tissue hypoxia and negative neural feedback with chronic decreased *α*-2 receptor responsiveness[16].

More broadly, other medications[12] can cause chronic nasal symptoms through a host of different mechanisms. Antihypertensive medications including *β*-blockers, angiotensin converting enzyme inhibitors, reserpine, calcium channel blockers, and methyldopa cause nasal congestion. A very bothersome side effect seen with *α*-receptor antagonists used for benign prostatic hyperplasia and phospophodiesterase-5 inhibitors for erectile dysfunction is nasal congestion. Aspirin and nonsteroidal anti-inflammatory drugs also may contribute to congestion, especially in patients with a history of nasal polyposis. Oral contraceptive pills also can cause congestion in some women. Personal observations by members of the roundtable (Roundtable Meeting, December 13, 2008, Washington, DC) suggest that topical eye drops such as *β*-blockers for glaucoma may also cause nasal symptoms.

Chronic nasal symptoms are associated with a variety of medical conditions, the full range of which is beyond the scope of this article (Table 2). For example, nasal congestion can be seen in distinct diseases such as hypothyroidism and chronic fatigue syndrome. Baraniuk and colleagues found that 46% of patients with chronic fatigue syndrome also have NAR and that 76% of these patients have ongoing nasal complaints[17]. Gastroesophageal reflux disease or laryngeal-pharyngeal reflux can both lead to chronic postnasal drip and other throat symptoms, and in severe cases, they can also cause nasal congestion[18]. Anatomical anomalies can also contribute to NAR. Adenoid hypertrophy, nasal septal deviation, and idiopathic turbinate hypertrophy or other structural abnormalities can cause chronic nasal obstruction with little relief from medications. Surgical intervention can be curative.

**Table 2:** Medical Conditions With NAR Symptoms

Metabolic
• Acromegaly
• Pregnancy
• Hypothyroidism
Autoimmune
• Sjogrens syndrome
• SLE
• Relapsing polychondritis
• Churg-Strauss syndrome
• Wegner's granulomatosis
Other
• Cystic fibrosis
• Kartagener's syndrome, ciliary dysfunction syndromes
• Sarcoidosis
• Immunodeficiency
• Amyloidosis
• Chronic fatigue syndrome
• Gastro-esophageal reflux and laryngopharyngeal reflux

Adapted from *Clin Allergy Immunol*. 2007;19:23-34 and *J Allergy Clin Immunol*. 2008;122(2 suppl):S1-S84.

Senile rhinitis is a clinically defined condition most common in the elderly and can lead to persistent watery rhinorrhea that may be worsened by food or environmental irritants. Gustatory rhinitis is the condition that causes anterior rhinorrhea and/or postnasal drip after eating, especially hot or spicy foods[19]. Gustatory rhinitis is more frequent in older individuals and overlaps with senile rhinitis. Both gustatory and senile rhinitis involve excessive secretions and are effectively treated with topical anticholinergic agents[19]. They can occur concomitantly and are often found in patients with classic VMR.

Atrophic rhinitis may occur as a primary, idiopathic syndrome with mucous gland atrophy or as a secondary syndrome after overzealous surgeries, with too many mucus-secreting tissues removed. Because mucus is required to restrict bacterial growth in mucous membranes, these patients have dry mucosa and often contract ozena. A few bacterial diseases (pseudomonas ozena and Klebsiella rhinoscleromatis) also can cause atrophic rhinitis[12].

CSF leak in patients with a history of craniofacial trauma or past facial/sinus surgeries must be considered when evaluating persistent rhinorrhea. Increased intracranial pressure can increase the risk of spontaneous CSF leak.

Historically, NAR variants have been divided into 2 groups based on nasal cytology: NARES and non-NARES. However, in this era, nasal cytology is rarely performed in clinical practice. When NARES was described,[20] patients had the same spectrum of symptoms as seen in AR and were noted to have eosinophilia in nasal secretions. However, skin testing and radioallergosorbent testing were negative. Thus, these eosinophilic patients all had the hallmarks of AR except for specific IgE antibodies. NARES patients characteristically respond well to topical nasal corticosteroids. Unfortunately, most clinicians no longer do nasal smears, and we do not know the relationship of NARES to the other forms of NAR.

"Occupational rhinitis" is not a separate category but simply designates the location where exposure to nasal triggers is encountered. In practice, many patients complain of occupational rhinitis. Rhinitis from occupation exposures can be caused by irritants and/or allergens. Noxious fumes, odors, and environmental irritants are classic provocateurs of occupational rhinitis, whereas colophony (solder fumes), chemicals, enzymes, and countless other manufacturing by-products can act as allergens to some exposed people[12,21]. With cleaner, more controlled environments, one might expect fewer occupational diseases in today's world, but patients often believe that work-related exposures are still a common cause of rhinitis. A good history, combined with inventive provocations (such as exposure to soldering fumes or detergent powder), helps define what condition or exposure is responsible for the patient's problems[21].

Chronic rhinosinusitis, with or without nasal polyps, is another cause of chronic rhinitis[22,23]. The symptoms of nasal congestion, postnasal drip with throat clearing and cough, facial pressure, headache, purulent drainage, and anosmia should lead the astute clinician to examine suspected sinus involvement. Because the inflammation in sinusitis may be infectious or immunologically mediated, these syndromes are usually not considered part of the nonallergic rhinitis syndromes[12,22-24].

Aspirin-exacerbated respiratory disease usually includes NAR as 1 of the clinical characteristics, along with sinusitis, nasal polyposis, asthma, and eosinophilia worsened with aspirin or nonsteroidal anti-inflammatory drug exposure[12]. The NAR seen in aspirin sensitivity is usually a form of NARES and may precede the development of the other manifestations of the syndrome.

## Nonallergic vasomotor rhinitis, which is now proposed to be called nonallergic rhinopathy

The most frequent form of NAR observed clinically is vasomotor rhinitis (VMR) or idiopathic rhinitis, characterized by persistent or intermittent nasal symptoms that can be triggered by environmental conditions that do not bother normal individuals. These triggers include the following: strong odors; exposure to cold air; changes in temperature, humidity, and/or barometric pressure; ingestion of alcoholic beverages; and changes in menstrual-related hormone levels. Patients can also have persistent symptoms in the absence of identified triggers. The diagnosis of VMR is primarily made by clinical history and exclusion of other known causes. If a patient has appropriate nasal symptoms (usually rhinorrhea, congestion, postnasal drip, headaches, facial pressure, throat clearing, and/or coughing) worsened or triggered by 1 or more of the environmental conditions noted above, then VMR is present. Concomitant ocular symptoms tend to be minimal, and the symptoms of nasal and palatal itch and also sneezing spells are not common.

Some patients with nonallergic rhinopathy have persistent nasal symptoms and no other recognized cause. These patients may or may not respond to the environmental conditions that trigger other symptoms in other patients. The clinical characteristics of these patients (predominantly female, adult onset, clinical symptoms, and response to treatment) are indistinguishable from VMR patients with recognized triggers. It is proposed that these patients also have VMR.

Unlike AR, VMR is usually adult onset and is not worsened by exposure to classic allergens such as pollen, house dust mite, dogs, or cats. A validated questionnaire has been created to help identify NAR patients[25]. Because VMR may be caused by shifts in temperature, humidity, and/or barometric pressure, patients may experience seasonal symptoms associated with changes in these climatic conditions experienced during the spring and fall. Seasonal VMR can, therefore, be confused with seasonal allergic rhinitis[26].

The diagnosis of VMR is based solely upon the patient's history of symptoms and their triggers, whereas the diagnosis of AR requires an appropriate history and confirmatory allergy testing--either positive relevant skin prick tests or radioallergosorbent tests. These 2 diseases are not mutually exclusive, and at least 60% of AR patients develop nasal symptoms in response to nonallergic environmental triggers. To have "pure" nonallergic rhinopathy, however, the patient must have negative relevant skin tests or in vitro allergen-specific antibody tests.

There are some limited data that histamine can be released by inhaling cold, dry air, which elicits cold-air-induced rhinitis in some sensitive patients. In a set of cold, dry air challenges, in vivo histamine release was observed with cold-air-induced rhinitis patients but not with other forms of NAR[27]. The importance of the histamine release to the development of the rhinitis symptoms was considered to be unrelated.

The epidemiologic predominance of females with VMR suggests that female hormones might play some role, but there is little supporting data. One study reported that 71% of patients with VMR were women compared with 62% of those with mixed rhinitis and 55% of those with AR[25]. These data are consistent with findings from other studies[1,2,15].

## Conclusions

Nonallergic rhinitis syndromes are heterogeneous and are often unrelated to each other. The 1 thing that these diseases have in common is that they are chronic, bothersome nasal conditions that do not involve allergic mechanisms. Few studies have explored etiologic causes, although it is recognized that sensory neuropeptides and cholinergic effecter discharges are involved in gustatory and senile rhinitis. There are data that neurosensory abnormalities play a role in nonallergic rhinopathy as well. However, until more specific subjective clinical characteristics and/or objective measures can better define and differentiate underlying causes for these disparate diseases, they will remain a murky group of overlapping syndromes.

## Note

Received grant/research support from GlaxoSmithKline (GSK), Sanofi-Aventis Pharmaceuticals, Schering Plough Corporation, Pfizer Inc, Meda Pharmaceuticals, AstraZeneca, Merck, and Alcon Laboratories, among others. The author is a consultant, or on an advisory board or the speakers bureau for GSK, Novartis, Genentech, Alcon, Meda, Cornerstone, Schering, Sepracor, and Sanofi-Aventis, among others.

Presented at a roundtable conference held in December 2008. The meeting was sponsored by the TREAT Foundation (Washington, DC) and supported through an unrestricted educational grant from Meda Pharmaceuticals. The funding company did not have any input into the development of the meeting or the proceedings, and the company was not represented at the roundtable meeting.
